# Supplementary material for: All-at-once RNA folding with 3D motif prediction framed by evolutionary information
Source: Nat Methods. 2025 Oct 3;22(10):2094–106. doi: 10.1038/s41592-025-02833-w (PMC12510878; doi:10.1038/s41592-025-02833-w)
Supplement: Supplementary file 3 — Source code to run CaCoFold-R3D. Data and results. [file 41592_2025_2833_MOESM3_ESM.gz › supplemental_material/results/CaCoFold-R3D/CaCoFold-R3D_Rfam/RF02136_HTT-AS1_3.R2R.sto.pdf]

RF02136_HTT-AS1_3

5′- U C U Y C G Y U G A G C C C C G C G C C U U C ● C C C G G G U G G G G C R C U G C G Y U ● Y C ● G ● G G Y C ● Y G G Y R
